# Supplementary material for: Construction of sublingual trilaminated Eszopiclone fast dissolving film for the treatment of Insomnia: Formulation, characterization and In vivo clinical comparative pharmacokinetic study in healthy human subjects
Source: PLoS One. 2022 Jun 9;17(6):e0266019. doi: 10.1371/journal.pone.0266019 (PMC9182695; doi:10.1371/journal.pone.0266019)
Supplement: Attachment — Submitted filename: Comments for PLOS_Jan 22.docx [file pone.0266019.s001.docx]

Comments for ‘Construction of Sublingual Trilaminated Eszopiclone Fast Dissolving Film for

Treatment of Insomnia: Formulation, Characterization and In-vivo Clinical Comparative

Pharmacokinetic Study in Healthy Human Subjects.’

Ref. No. PONE-D-21-37028

I thank the editor for giving me an opportunity to review this manuscript. Work done is good but many grammatical errors, unnecessary making word capital is seen which should be avoided. It requires major revision.

Few comments are as follows:

1) pH word in abstract should be corrected.
2) Heading for 2.2.2, what is tril?
3) Please explain rational for selections of polymer concentration. It is based on Literature search or placebo trials.
4) 2.2.4 : In which layer and when drug should be added is not mentioned.
5) 2.2.5, sandwich approach is good but is drug migration occurs from middle to end layer?
6) P from petri plates should be small at many places.
7) Is for testing in volunteers any ethical approval taken?
8) Why ariprirazole is selected as IS?
9) Line 276, page 14. Gives characteristics of buccal film. Is film described in this manuscript buccal or dissolving?
10) Line 317, page 16, says results are due to polysaccharide properties of pullulan. But maximum good taste is shown by F4 having sodium alginate as polymer. Explain.
11) Figure 1 showed DSC thermogram of excipients and 1 physical mixture. It is physical mixture with which excipient? For better understanding of interaction, authors should show physical mixtures of drug with all other polymers.
12) LC-MS/MS chromatogram of drug and IS should be shown.
13) Statistical analysis is explained but not seen in discussion , in figures please include.
