# Supplementary material for: Construction of sublingual trilaminated Eszopiclone fast dissolving film for the treatment of Insomnia: Formulation, characterization and In vivo clinical comparative pharmacokinetic study in healthy human subjects
Source: PLoS One. 2022 Jun 9;17(6):e0266019. doi: 10.1371/journal.pone.0266019 (PMC9182695; doi:10.1371/journal.pone.0266019)
Supplement: Attachment — Submitted filename: Peer Reviewer 2 comments.docx [file pone.0266019.s002.docx]

**Peer Reviewer: 2**

- **The main concern is that films disintegrated over 10 minutes, much longer than fast dissolving oral films.**

**Response:**

It is typing error and the correct time is (**sec**). The correct time has been written in the revised manuscript.

The disintegration time of all formula were found to be between 10 to 40 sec so it is called fast dissolving film. And seen in Table 2 (In vitro evaluation of medicated fast dissolving films (FDF)), page 14 in the main manuscript.

1. **There are paragraphs in the Introduction that do not cite references such as the last paragraph in page 3. Also, there is an incomplete sentence on page 3 line 60, and line 68, “better” should be replaced with “bitter”.**

**Response:**

a. We revised the manuscript and the references were added to uncited paragraph and highlighted with yellow color. Additional references were added to other paragraphs which lack of references such as line 154,156,159.
b. The incomplete sentence was revised and completed.
c. **“**Better**”** word has been replaced with **“**bitter**”** in line 69.
2. **Could the authors please specify the calibration curve characteristics of eszopiclone for UV measurements on page 8.**

**Calibration table**

| **Abs at λ_max_ 304** | **Concentration µg/ml** |
| --- | --- |
| **0** | **0** |
| **0.197** | **6** |
| **0.281** | **9** |
| **0.359** | **12** |
| **0.447** | **15** |
| **0.521** | **18** |
| **0.607** | **21** |
| **0.696** | **24** |
| **0.764** | **27** |
| **0.859** | **30** |
| **0.934** | **33** |

Abs at λmax 304

| conc µg/ml | std calibration curve |
| --- | --- |
| 0 | 0 |
| 6 | 0.197 |
| 9 | 0.281 |
| 12 | 0.359 |
| 15 | 0.447 |
| 18 | 0.521 |
| 21 | 0.607 |
| 24 | 0.696 |
| 27 | 0.764 |
| 30 | 0.859 |
| 33 | 0.934 |

**Figure 4 : calibration curve of eszopiclone**

This figure was added to line 146 page 8 and highlighted.

**3. Were ethics obtained to test the taste of films in volunteers and what were exclusion and inclusion criteria?**

**Response:**

The affirmed protocol set by the ethics committee of the Faculty of Pharmacy, Cairo University, Egypt with serial number: PI (2313) and the date of this approval (26 / 11 /2018).

Inclusion criteria:

•	Human Subjects from 18 to 55 years of age (inclusive of both).

•	Subjects within the BMI range from 18 to 35 kg/m2.

•	Subjects with normal range of vital signs (Blood Pressure, Pulse Rate, Respiratory Rate and Body Temperature).

•	Subjects with normal Medical and Surgical history without illness within the last 4 weeks prior to start of the study.

•	Subjects with normal functioning of Cardiovascular, Respiratory, Gastrointestinal, Nervous System, Musculoskeletal, Vascular, GenitoUrinary, Endocrine/Metabolic systems.

•	Subjects with normal Lymph nodes, head, neck, eyes, ears, nose, throat and skin.

•	Subjects with normal laboratory investigations

•	Subjects able to communicate effectively.

•	Subjects willing to provide informed consent and adhere to the protocol requirements.

•	Urine analysis for narcotic drug (for all subjects) / Pregnancy test (for females) will be conducted prior to each phase.

Exclusion Criteria:

•	Contraindications or Hypersensitivity to Eszopiclone or related group of drugs.

•	History or presence of any medical condition or disease according to the opinion of the physician.

•	History or presence of significant alcoholism or drug abuse in the past one year.

•	History or presence of significant smoking (more than 09 cigarettes/day or consumption of tobacco products and refusal to restrain from smoking or consumption of tobacco products for 48.00 hours before dosing until checkout).

•	History or presence of significant renal or hepatobiliary problems.

•	History or presence of significant asthma, urticaria or other allergic reactions.

•	History or presence of significant gastric and/or duodenal ulcers.

•	Difficulty in donating blood.

•	Difficulty in swallowing film coated tablet or capsules.

•	Systolic blood pressure less than 90 mm Hg or more than 140 mm Hg.

•	Diastolic blood pressure less than 60 mm Hg or more than 100 mm Hg.

•	Pulse rate less than 50/minute or more than 100/minute.

•	Use of any prescribed medication during last two weeks or OTC medicines or medicinal products during the last one week preceding the first dosing.

•	Major illness during 3 months before screening.

•	Subjects who have been on an abnormal diet during the four weeks before screening.

•	Participation in a drug research study within past 3 months.

•	Donation of blood in the past 3 months before screening.

•	Refusal to abstain from water for at least one hour prior to study drug administration and for at least one hour post dose.

•	Refusal to abstain from food for at least ten hours before dosing and for at least 3 - 4 hours’ post dose.

•	Refusal to abstain from alcohol or methylxanthine-containing beverages or foods (coffee, tea, carbonated drinks, chocolate) from 2 days prior to dosing till last sample collection.

•	HIV positive.

•	Anti-HCV antibodies positive.

•	Found positive in the Urine drugs of abuse done at the time of check-in at the beginning of each period.

•	Evidence of an uncooperative attitude.

**4. It is claimed on page 11, Line 225 that the LC-MS data were validated, where is the data or reference? What were the limit of detection and limit of quantification?**

**Response:**

LC-MS were validated as seen in the calibration curve:

Calibration Curve

| Conc (ng/mL) | PAR |
| --- | --- |
| 0.5 | 0.071143 |
| 5 | 0.0804657 |
| 10 | 0.162718 |
| 15 | 0.248214 |
| 25 | 0.341292 |
| 50 | 0.537349 |
| 75 | 1.00974 |
| 100 | 1.48152 |

| **conc (ng/ml)** | **peak area of IS** | **peak area of drug** | **PAR** |
| --- | --- | --- | --- |
|  | **413949** | **0** | **0** |
| **0.5** | **418411** | **29767** | **0.07114297** |
| **5** | **456679** | **36747** | **0.08046571** |
| **10** | **408909** | **66537** | **0.16271836** |
| **15** | **437259** | **108534** | **0.24821444** |
| **25** | **497676** | **169853** | **0.34129233** |
| **50** | **489784** | **263185** | **0.53734912** |
| **75** | **401758** | **405673** | **1.00974467** |
| **100** | **441067** | **653450** | **1.48152095** |

The limit of detection is 0.05 ng/ml and the limit of quantification is 0.5 ng/ml.

**5. Please use past tense for line 257.**

**Response:** past tense was used and corrected in the revised manuscript.

**6. Line 284, please cite a reference.**

**Response:** the reference was added to line 284.

**7. How did the volunteers hold a film for 10 minutes in the mouth, did they chew them and then swallow?**

**Response:** our formula was prepared as fast dissolving film to achieve higher plasma concentration in lower tmax and also to decrease residence time in the mouth to enhance patient compliance for this very bitter drug. The disintegration time of all formula were found to be between 10 to 40 seconds. A written mistake of the time was correct to sec. The correct time has been written in the revised manuscript in Table 2 (In vitro evaluation of medicated fast dissolving films (FDF)), page 14.

**8. Did the authors obtain GMP grades of active ingredient and excipients to prepare the oral films for volunteers? Who supplied these?**

**Response:**

In the material section point 2.1 line 76 page 4. Chemicals and Excipients we described where we obtained each material and each chemical used was supplied with material safety data sheet (MSDS).

**9. A statistical method is described in the Method section but the results are not presented.**

**Response:**

All statistical results are attached with the revised manuscript.

A one-way analysis of variance (ANOVA) followed by the least significant difference (LSD) as a post hoc test was applied; using SPSS program version 17 software. The differences were considered significant if P<0.05. The column effluent was detected spectrophotometrically at 304 nm. Retention time for aripiprazole was 1.929 min which is well differentiated from the peak of the eszopiclone drug which has retention time 1.865 min as shown in figure (3b) which also revealed that there were no peaks due to formula compounds that might interfere with the assay. Calibration curve were constructed for eszopiclone with an equation that best describe the curve is (y = 0.0137 x – 0.0102	) as y is represented by peak area and x for the concentration in (ng/ml) (r2 = 0.9774	) as shown in figure (3d).The limit of detection and the limit of quantitation were 0.05 and 0.5 (ng/ml) respectively.

**Discussion**: When we compare the reference tablet with the three fast dissolving films containing sodium alginate or xanthan gum or pullulan as polymer to study its effect on Cmax , AUC 0-24 and AUC 0-INF. A significant difference (p-value < 0.05) was found between the reference and F4 which contain sodium alginate as polymer in all parameters tested as seen in the sheet attached with our response. Tmax is decreased in F4 formulae which contain sodium alginate as polymer and it was 1.063 h for F4 while 2.25 h only for commercial tablet . Relative bioavailability is increased 1.6 fold for F4, 1.2 fold for F2 and 1.1 fold for F6.

1.	Ponrasu, T., et al., *Fast Dissolving Electrospun Nanofibers Fabricated from Jelly Fig Polysaccharide/Pullulan for Drug Delivery Applications.* Polymers, 2021. **13**(2): p. 241.

2.	Abu-Huwaij, R., et al., *Formulation and in vitro evaluation of xanthan gum or carbopol 934-based mucoadhesive patches, loaded with nicotine.* AAPS PharmSciTech, 2011. **12**(1): p. 21-27.

3.	Phaechamud, T. and G.C. Ritthidej, *Formulation variables influencing drug release from layered matrix system comprising chitosan and xanthan gum.* AAPS PharmSciTech, 2008. **9**(3): p. 870-877.

4.	Senturk Parreidt, T., K. Müller, and M. Schmid, *Alginate-Based Edible Films and Coatings for Food Packaging Applications.* Foods (Basel, Switzerland), 2018. **7**(10): p. 170.

5.	Duckworth, P.F., et al., *Alginate films augmented with chlorhexidine hexametaphosphate particles provide sustained antimicrobial properties for application in wound care.* Journal of materials science. Materials in medicine, 2020. **31**(3): p. 33-33.

6.	Zulkiflee, I. and M.B. Fauzi, *Gelatin-Polyvinyl Alcohol Film for Tissue Engineering: A Concise Review.* Biomedicines, 2021. **9**(8): p. 979.

7.	Karthikeyan, D., S. Sri, and C.S. Kumar, *Development of Fast Dissolving Oral Film Containing of Rizatriptan Benzoate as an Anti Migraine Medication.* Indo American Journal of Pharmaceutical Research, 2013. **3**(3): p. 2642-2654.
